# Enhanced Nitric Oxide Sensing Performance of Conjugated Polymer Films through Incorporation of Graphitic Carbon Nitride

**DOI:** 10.3390/ijms24021158

**Published:** 2023-01-06

**Authors:** Proscovia Kyokunzire, Ganghoon Jeong, Seo Young Shin, Hyeong Jun Cheon, Eunsol Wi, Minhong Woo, Trang Thi Vu, Mincheol Chang

**Affiliations:** 1 Department of Polymer Engineering, Graduate School, Chonnam National University, Gwangju 61186, Republic of Korea; 2 Alan G. MacDiarmid Energy Research Institute, Chonnam National University, Gwangju 61186, Republic of Korea; 3 School of Polymer Science and Engineering, Chonnam National University, Gwangju 61186, Republic of Korea

**Keywords:** conjugated polymers, poly(3-hexylthiophene), graphitic carbon nitride (g-C₃N₄), charge transfer, NO gas sensor

## Abstract

Organic field-effect transistor (OFET) gas sensors based on conjugated polymer films have recently attracted considerable attention for use in environmental monitoring applications. However, the existing devices are limited by their poor sensing performance for gas analytes. This drawback is attributed to the low charge transport in and the limited charge–analyte interaction of the conjugated polymers. Herein, we demonstrate that the incorporation of graphitic carbon nitride (g-C₃N₄) into the conjugated polymer matrix can improve the sensing performance of OFET gas sensors. Moreover, the effect of graphitic carbon nitride (g-C₃N₄) on the gas sensing properties of OFET sensors based on poly(3-hexylthiophene) (P3HT), a conjugated polymer, was systematically investigated by changing the concentration of the g-C₃N₄ in the P3HT/g-C₃N₄ composite films. The obtained films were applied in OFET to detect NO gas at room temperature. In terms of the results, first, the P3HT/g-C₃N₄ composite films containing 10 wt.% g-C₃N₄ exhibited a maximum charge carrier mobility of ~1.1 × 10^−1^ cm^2^ V^−1^ S^−1^, which was approximately five times higher than that of pristine P3HT films. The fabricated P3HT/g-C₃N₄ composite film based OFET sensors presented significantly enhanced NO gas sensing characteristics compared to those of the bare P3HT sensor. In particular, the sensors based on the P3HT/g-C₃N₄ (90/10) composite films exhibited the best sensing performance relative to that of the bare P3HT sensor when exposed to 10 ppm NO gas: responsivity = 40.6 vs. 18.1%, response time = 129 vs. 142 s, and recovery time = 148 vs. 162 s. These results demonstrate the enormous promise of g-C₃N₄ as a gas sensing material that can be hybridized with conjugated polymers to efficiently detect gas analytes.

## 1. Introduction

Environmental pollution detection, particularly toxic gas detection, is critical for industry, agriculture, and public health [1,2,3,4,5,6,7]. Many serious issues caused by various gaseous pollutants are emerging rapidly because of rapid industrial growth and an increase in the number of vehicles [8]. The gaseous pollutant nitric oxide (NO) is primarily produced as a byproduct of combustion processes in power plants, waste incinerators, and internal combustion engines [9,10]. NO gas is not only a precursor of acid rain but is also the cause of ozone depletion. In the presence of excess oxygen, NO is easily oxidized to nitrogen dioxide, and it is known that frequent exposure to NO and NO_2_ gases leads to numerous medical conditions, including itchiness, diabetes, heart failure, asthma, pulmonary inflammation, and death. Therefore, the detection of these toxic gases is crucial for maintaining safe operating environments in the agriculture, industry, and healthcare domains [11,12,13,14,15]. Consequently, the development of real-time monitoring strategies for NO gas and other pollutants is an urgent requirement, and it necessitates the use of sensing materials that are low-cost, robust, easily processable, and sensitive with a fast response time [16,17,18,19]. In view of these requirements, conjugated polymers have attracted considerable interest among various gas sensing materials and have emerged as competitive alternatives to conventional inorganic semiconductors owing to their extensive material diversity, flexibility, compatibility with large-scale processing techniques, light weight, operation at room temperature, and tailorable structures [20,21,22,23,24].

To date, several types of gas sensors that are extremely sensitive to noxious gases, with detection limits as low as 0.1 ppm, have been proposed and developed. However, several of these sensors offer poor responses or require high working temperatures (60–300 °C) [25]. Examples of such devices include metal oxide sensors [26,27], resistive sensors [28,29,30], amperometric sensors [31,32], and carbon nanotubes [33,34]. In particular, inorganic metal oxide-based sensors can only exhibit limited selectivity, and a high temperature is generally required for their operation [26], hindering their use in practical sensor applications. Furthermore, their high power requirements and consumption limit their portability and real-time sensing applications [35].

Recently, organic field-effect transistors (OFETs), three-terminal electronics using organic semiconductors (OSCs) as an active layer, have attracted great attention in numerous research areas [36]. On the technological side, OFETs are considered to be a key component of organic integrated circuits for use in flexible smart cards, low-cost radio-frequency identification tags, and organic active-matrix displays [37]. On the scientific side, OFETs are also an effective unit to test novel OSCs and investigate organic electronics [38]. During the past several decades, the design and synthesis of innovative OSCs, the introduction of new device fabrication methods, the implantation of suitable gate insulator layers, interface engineering, crystal engineering, etc., have contributed to significant advances in high-performance OFETs [36,39,40]. Owing to these advances, OFETs have become comparable to or have even surpassed amorphous silicon thin-film transistors in both performance and cost [41]. Therefore, considering the intrinsic merits of OSCs and their good biocompatibility over their inorganic counterparts makes OFETs promising candidates not only for physical and chemical sensors but also for biosensors [41,42].

In view of the above, transistor-type sensors, in particular conjugated-polymer-based organic field-effect transistor (OFET) gas sensors, have attracted significant research interest owing to their ease of fabrication, room-temperature operation, diverse fabrication materials, tailorable structures, and signal amplification abilities, which have opened new possibilities for the accurate detection of trace noxious gases at industrial sites [41,43,44]. OFET-type sensors detect gases on the basis of the interactions of gas molecules with charge carriers in the channel region [45]. As gas analytes permeate through organic semiconducting films, they interact with polymer molecules through the Van der Waals force, dipole–dipole interaction, and dipole–charge interaction, resulting in changes in the charge carrier mobility, threshold voltage, and on/off current ratio of the OFETs [23,41,42]. However, the OFETs based on conjugated polymers have exhibited insufficient sensing performance, particularly in the detection of oxidizing gases such as NO, NO_2_, and SO_2_ [46,47].

Two-dimensional (2D) polymer-based graphitic carbon nitride (g-C₃N₄) is an emerging material in the broad class of carbon materials, and it has garnered remarkable scientific attention for its use in various applications [48]. This is because of its peculiar properties, such as its ordered units of tri-s-triazine rings bonded together with the nitrogen atom in the layered structure, which provide a large surface area [49,50,51,52]. In contrast to graphene, g-C_3_N_4_ is a conjugated organic semiconductor composed of Van der Waals sheets of sp2-hybridized carbon and nitrogen atoms [53,54,55]; it is an abundant, low-cost, and simple-to-manufacture nanomaterial that is suitable for large-scale use [56]. Furthermore, the remarkable physiochemical features of 2D g-C₃N₄, such as its good biocompatibility, low density, high thermal and chemical stability, appropriate bandgap, and high electron mobility, make it a good candidate with potential for use in diverse domains such as gas sensing, energy storage, catalysis, optoelectronics [57,58,59,60,61,62,63], dye degradation [64], and water splitting [65]. In particular, owing to its abundant amine functional groups and inherent porous structure, g-C₃N₄ can act as a potential sensor material with a large surface area and an abundance of active sites that would be suitable for realizing various sensor applications [66,67]. Because of these merits, researchers have attempted to hybridize g-C₃N₄ with various inorganic materials to develop gas sensor applications [68]. For example, Ritu Malik et al. integrated g-C₃N₄ with In-SnO₂ to design an ultrasensitive toluene gas sensor that can operate at low temperatures [69], and they also developed a fast-response relative humidity sensor using a blended composite consisting of g-C₃N₄ and In-SnO₂ [70]. Ibrahim et al. incorporated palladium (Pd) nanoparticles into g-C₃N₄ to obtain Pd/g-C₃N₄, and their results indicated that this material allowed for efficient hydrogen gas sensing [71]. Shaolin Zhang et al. prepared g-C₃N₄/graphene nanocomposites to improve the NO_2_ gas sensing performance of a pure graphene sensor at room temperature. The developed nanocomposite sensor exhibited better recovery and responded twice as fast as the pure graphene sensor [62]. However, to our knowledge, few attempts have been made to integrate g-C₃N₄ with conjugated polymers to realize improved OFET gas sensors.

In this study, we demonstrate that poly(3-hexylthiophene) (P3HT)/g-C₃N₄ composite films exhibited improved NO gas sensing performance compared to bare P3HT films. The concentration of g-C₃N₄ in the P3HT/g-C₃N₄ composite films was varied to systematically study its effects on the morphology, molecular ordering, and electrical properties of the films using ultraviolet-visible (UV-Vis) spectroscopy, optical microscopy (OM), atomic force microscopy (AFM), Raman spectroscopy, X-ray photoelectron spectroscopy (XPS), and charge mobility measurements. Importantly, the P3HT/g-C₃N₄ composite films with 10 wt.% g-C₃N₄ exhibited a maximum charge carrier mobility of ~1.1 × 10^−^^1^ cm^2^ V^−^^1^ S^−^^1^, which was approximately two times higher than that of bare P3HT films. Furthermore, the NO gas sensing behaviors of the P3HT/g-C₃N₄ composite films were investigated thoroughly. To this end, an OFET sensor based on the P3HT/g-C₃N₄ (90/10 *w/w*%) composite was fabricated, and the responsivity of this sensor was found to be approximately two times higher than that of a bare P3HT OFET sensor. In addition, the sensor exhibited improved response (129 s) and recovery (148 s) times compared to those (142 and 162 s, respectively) of the bare P3HT OFET sensor.

## 2. Results and Discussion 

### 2.1. Synthesis of P3HT/g-C₃N₄ Composite Films

**Figure 1:**
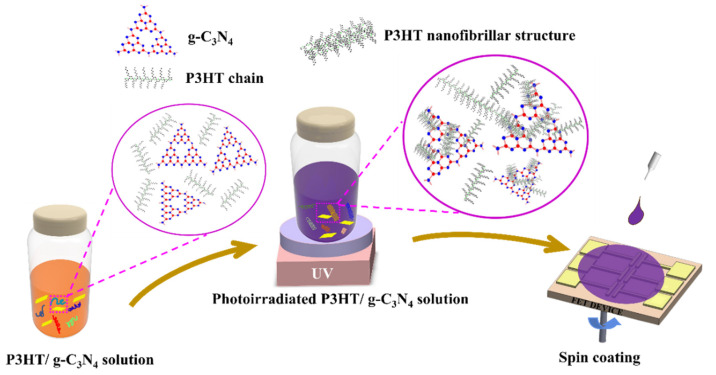
Schematic illustration of the preparation of P3HT/g-C_3_N_4_ composite films by means of photoirradiation and the subsequent spin-coating of the composite solutions onto an FET device.

Figure 1 presents a schematic illustration of P3HT/g-C₃N₄ composite film fabrication employing a UV-photoirradiation-based approach. The P3HT/g-C₃N₄ composite solutions were prepared by mixing a P3HT solution and a g-C₃N₄ dispersion in chloroform and then exposing the composite solution to UV light at room temperature to improve the dispersion stability of the g-C₃N₄ [72]. The conjugated polymer chains (P3HT in solution) were photoexcited upon exposure to UV irradiation, resulting in enhanced π-orbital overlap along the polymer backbone and the subsequent planarization of the chains, which allowed for the delocalization of π-electrons and resulted in the self-assembly of P3HT chains [22,73,74,75]. Because g-C₃N₄ is characterized by a stacked 2D structure and van der Waals interactions between the g-C₃N₄ sheets, and the –NH_2_, –NH, or –OH groups on its edge can act as active sites to initiate the nucleation and growth of crystalline P3HT nanofibrillar structures, g-C₃N₄ facilitated the self-assembly of P3HT chains under UV light irradiation [76]. The g-C₃N₄ particles that were in contact because of Van der Waals or electrostatic interactions with the P3HT nanofibrillar structures were well dispersed in the composite solution [72,77]. Consequently, the P3HT/g-C₃N₄ composite films prepared using the corresponding solutions exhibited good distributions of P3HT/g-C₃N₄ within the P3HT matrix.

### 2.2. Effect of g-C₃N₄ Content on Film Morphology

**Figure 2:**
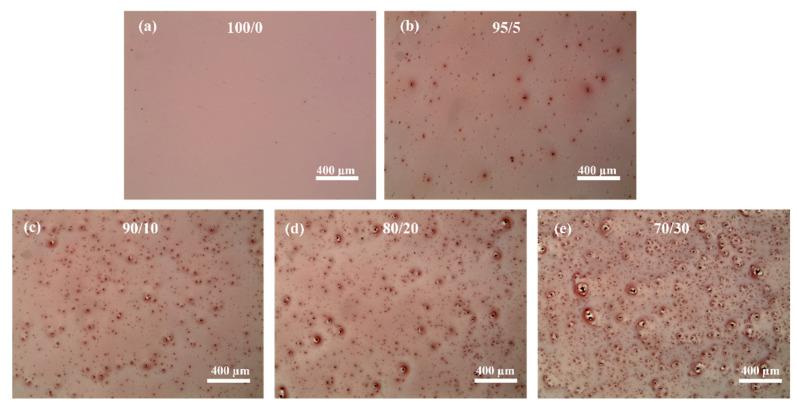
OM images of films deposited using photoirradiated solutions of (**a**) bare P3HT (100/0), (**b**) P3HT/g-C₃N₄ (95/5), (**c**) P3HT/g-C₃N₄ (90/10), (**d**) P3HT/g-C₃N₄ (80/20), and (**e**) P3HT/g-C₃N₄ (70/30).

OM and SEM images of the bare P3HT film and the P3HT/g-C₃N₄ composite films that were spin-coated using bare P3HT and the aforementioned P3HT/g-C₃N₄ blends, respectively, were obtained to investigate the miscibility between P3HT and g-C₃N₄ (Figure 2 and Figure S1). The bare P3HT film exhibited a homogeneous and smooth morphology owing to the good solubility of P3HT in the chloroform solution [78]. The g-C₃N₄ particles appeared to be well dispersed throughout the film up to a concentration of 10% (Figure 2b,c). However, the SEM and OM images of the surfaces of the composite films prepared by adding more than 10% g-C₃N₄ exhibited morphologies with many large g-C₃N₄ aggregates, as illustrated in Figure 2d,e and Figure S1e,d. As depicted in Figure S2, although g-C₃N₄ was well dispersed in the initial solution after sonication, it precipitated completely after 12 h. This was attributed to the weak dispersibility of g-C₃N₄ in organic solvents owing to its strong van der Waals attraction (π–π stacking) of sp_2_ carbons, which resulted in re-agglomeration during film deposition [72,76,79]. Nevertheless, the P3HT/g-C₃N₄ composite films fabricated using the corresponding photoirradiated solutions remained homogenous for 12 h and exhibited better P3HT/g-C₃N₄ distributions than those of the pristine P3HT/g-C₃N₄ films (Figure S3).

**Figure 3:**
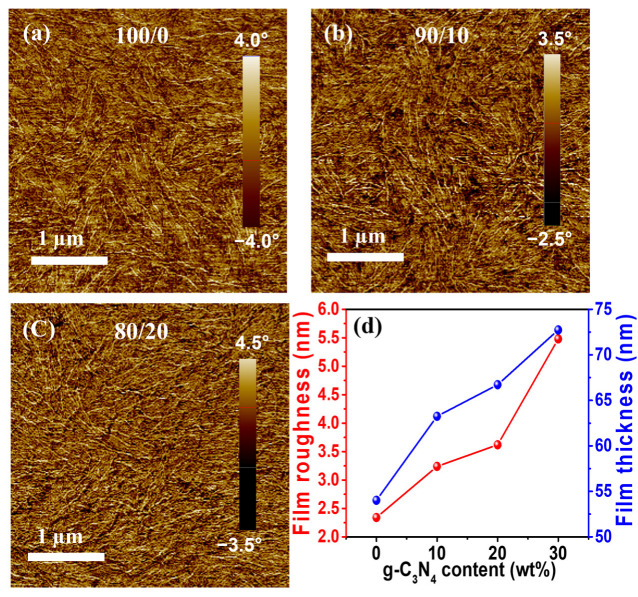
AFM images of (**a**) bare P3HT (100/0), (**b**) P3HT/g-C₃N₄ (90/10), and (**c**) P3HT/g-C₃N₄ (80/20) composite films. (**d**) Thickness and roughness of P3HT/g-C₃N₄ composite films with different g-C₃N₄ contents (0, 10, 20, and 30 wt.%).

AFM was used to investigate the influence of g-C₃N₄ incorporation on the morphology of the resulting P3HT/g-C₃N₄ composite films, as shown in Figure 3. The film that was spin-coated with a solution containing photoirradiated bare P3HT exhibited distinct P3HT nanowires (Figure 3a), which was consistent with the results in the literature [74]. In the P3HT/g-C₃N₄ composite films, increased formation of P3HT nanowires (NWs) was observed (Figure 3b,c), which supported the formation of P3HT NWs facilitated by g-C_3_N_4_ during the photoirradiation of the composite solutions [73,74,75]. The number of P3HT nanowires increased gradually with the g-C₃N₄ content until 20 wt.% (Figure 3c). AFM measurements could not successfully visualize the morphologies of the photoirradiated P3HT/g-C₃N₄ composite films fabricated with g-C₃N₄ concentrations higher than 20% because of the large topographical gaps between the P3HT and g-C₃N₄ domains [73]. Moreover, the surface roughness and thickness of the P3HT/g-C₃N₄ composite films increased, as shown in Figure 3d. For instance, the roughness values of the P3HT/g-C₃N₄ composite films fabricated using the 100/0, 90/10, 80/20, and 70/30 blends increased to 2.3, 3.2, 3.6, and 5.5 nm, respectively. The increased roughness was ascribed to the increased quantity of the P3HT NWs in the resulting films [74,80]. Similarly, the film thickness tended to increase from ~54.0 to ~72.7 nm when the g-C₃N₄ content in the P3HT/g-C₃N₄ composite films was increased from 0 to 30%.

### 2.3. Photophysical Properties of the P3HT/g-C₃N₄ Composite Films

**Figure 4:**
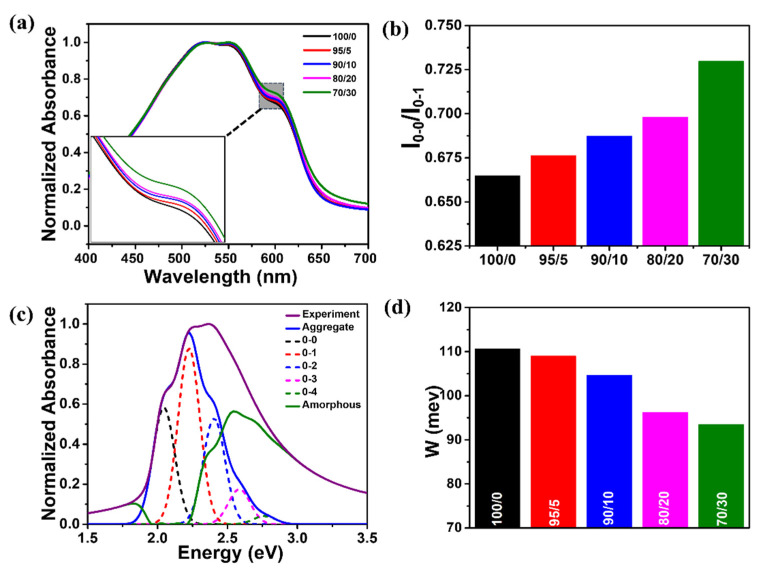
(**a**) Normalized UV-Vis absorption spectra of P3HT/g-C₃N₄ composite films according to g-C₃N₄ content (0, 5, 10, 20, and 30 wt.%). (**b**) Intensity ratios of (0-0) and (0-1) transitions of the corresponding composite films. (**c**) Absorption spectrum of P3HT/g-C₃N₄ (90/10) composite film deconvoluted by Spano analysis using Equation (1). The green line indicates the spectrum of amorphous P3HT chains, and the blue solid line depicts the spectrum of the P3HT aggregates in the film. (**d**) Calculated exciton bandwidth (W) of the P3HT/g-C₃N₄ composite films with different g-C₃N₄ contents.

To elucidate the intramolecular ordering of the P3HT polymer chains in the P3HT/g-C₃N₄ composite films, the films were characterized using a combination of UV-Vis absorption spectroscopy and quantitative modeling. Figure 4a shows the UV-vis absorbance spectra of the bare P3HT and P3HT/g-C₃N₄ composite films. These spectra highlight two features of the films: A higher-energy band (π–π* intraband transition) at ~520 nm, which correlates to disordered single-polymer chains, and lower-energy features (i.e., (0–1) transition at ~552 nm and (0–0) transition at ~605 nm), which correlate to the well-ordered aggregates. Relative to the higher-energy band, the lower-energy bands developed as the g-C₃N₄ content increased. This reflected an increase in the proportion of ordered aggregates formed through favorable intermolecular interactions between the P3HT chains upon the addition of g-C₃N₄ [75,81,82,83]. In addition, I_0-0_/I_0-1_, the ratio of the (0–0) and (0–1) peak intensities, increased as the amount of g-C₃N₄ increased from 0 to 30 wt.% (Figure 4b). Higher I_0-0_/I_0-1_ values signify an improvement in the degree of intramolecular ordering in single-P3HT chains [81,84]. P3HT aggregates were formed as weakly coupled H-types, as indicated by the intensity ratios of the films, which were less than 1 [82,85]. The intramolecular ordering of P3HT is related to the exciton bandwidth (W) [86]. Static absorption spectroscopy coupled with the Spano model, which explains the theoretical contribution of the well-ordered P3HT aggregates to absorption, was used to calculate the exciton bandwidth (W); the Spano model was applied to the experimental spectra, as shown in Figure 4c [74,82,87]. The intrachain ordering of the P3HT chains comprising the aggregates was found to be correlated to the W value obtained using Equation (1) [81]:

(1) $$A\propto \underset{m=0}{\sum }\left(\frac{e^{-s}S^{m}}{m!}\right)\times {\left(1-\frac{We^{-s}}{2E_{p}}G_{m}\right)}^{2}\times exp\left(-\frac{{\left(E-E_{0-0}-mE_{p}-1/2WS^{m}e^{-s}\right)}^{2}}{2\sigma^{2}}\right)$$

where *A* is the theoretical absorbance of the P3HT aggregates as a function of the photon energy (E), *S* is the Huang–Rhys factor (~1.0), *Ep* is the intermolecular vibrational energy of the symmetric vinyl stretching mode (~0.18 eV), *σ* is the Gaussian linewidth, *Gm* is a constant that depends on the vibrational level (m) (e.g., *m* = 0 for the (0-0) transition defined by *Gm* = ∑*n*(≠*m*)*S^n^*/*n*!(*n* − *m*)), and n is the vibrational quantum number [82]. As shown in Figure 4d, the W values of the P3HT/g-C₃N₄ composite films were lower than those of the bare P3HT films. A further decrease in W was observed as the g-C_3_N_4_ content increased, which indicated an enhancement in the intramolecular ordering of the P3HT chains [73]. For instance, the W value of the P3HT/g-C₃N₄ composite film containing 5 wt.% g-C₃N₄ was calculated to be ~109 meV, and it decreased to ~93 meV when the g-C₃N₄ concentration in the composite film was increased to 30 wt.%.

### 2.4. Interfacial Interactions between P3HT and g-C₃N₄

**Figure 5:**
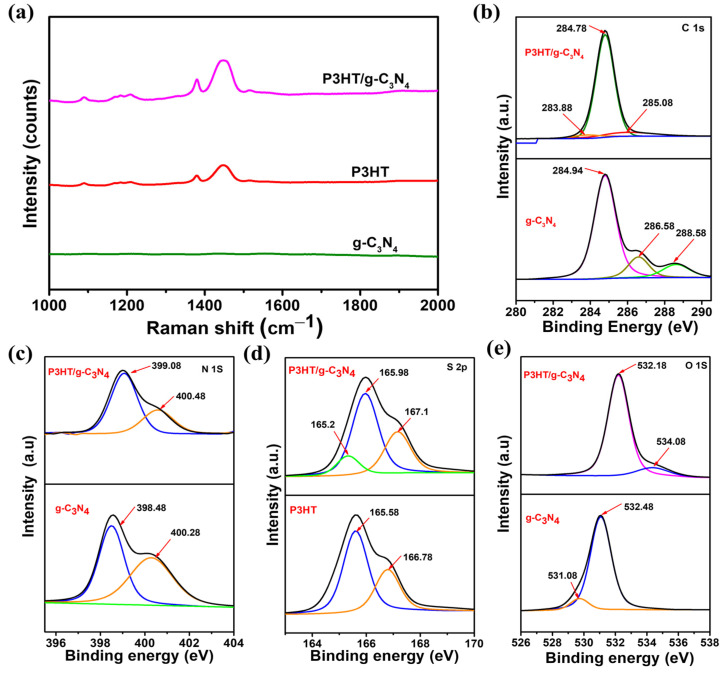
(**a**) Raman spectra of g-C₃N₄, P3HT, and P3HT/g-C₃N₄ composite films. XPS survey spectra of (**b**) C 1s for g-C₃N₄ and P3HT/g-C₃N₄, (**c**) N 1s for g-C₃N₄ and P3HT/g-C₃N₄, (**d**) S 2p for P3HT and P3HT/g-C₃N₄, and (**e**) O 1s for g-C₃N₄ and P3HT/g-C₃N₄.

Figure 5a shows the Raman spectra of the g-C₃N₄, P3HT, and P3HT/g-C₃N₄ composite samples obtained at an excitation wavelength of 514 nm to further study the intramolecular ordering of the P3HT chains in the prepared P3HT/g-C₃N₄ composite films. g-C₃N₄ did not present any obvious Raman signals. P3HT presented strong Raman signals at 1379 and 1445 cm^−^^1^. P3HT/g-C₃N₄ presented strong Raman signals at 1380 and 1447 cm^−^^1^, which were ascribed to C-C skeletal stretching and C=C ring stretching, respectively, and are sensitive to π-electron delocalization (conjugation length), namely the intramolecular ordering of P3HT molecules [73,88]. The Raman intensity of the C=C mode of the P3HT/g-C₃N₄ composite under 514 nm excitation increased by two times compared to that of bare P3HT, and this increase was induced by a preresonance Raman effect, leading to an increase in the intensity of the Raman peaks. This result indicated that more ordered and longer conjugated segments (i.e., enhanced intramolecular ordering) existed in the P3HT/g-C₃N₄ composite system [88].

The interaction between P3HT and g-C₃N₄ was further verified using high-resolution X-ray photoelectron spectroscopy (XPS), as shown in Figure 5b–e. Both g-C₃N₄ and the P3HT/g-C₃N₄ composite contained C, N, and O, and the P3HT/g-C₃N₄ composite contained S as well. The core-level C 1s XPS spectrum is shown in Figure 5b. The g-C₃N₄ peak observed at the binding energy of 284.94 eV was attributed to the C-C coordination of the amorphous graphitic carbon atom [89,90]. The other two peaks at 286.58 and 288.58 eV were assigned to the C-NH_2_ bond and the nitrogen bond containing the aromatic ring in the sp^2^ hybridized N=C-N carbon atom, respectively [48]. The peaks of the P3HT/g-C₃N₄ composite film at 285.08 and 284.78 eV corresponded to the C atom in the alkyl chain and the thiophene ring, respectively. The difference between the C 1s binding energy values of g-C₃N₄ and P3HT/g-C₃N₄ suggested that the P3HT was stacked and interacted with g-C₃N₄ rather than being simply mixed with it [89]. Figure 5c shows the deconvoluted fitting characteristic peaks in N 1s spectra at 398.4 and 400.28 eV. The peaks at 398.4 and 400.28 eV were assigned to the sp^2^ hybridized C-N-C nitrogen atom present in the triazine structural unit and the N-(C)_3_ tertiary nitrogen-linking structure in g-C₃N₄, respectively [48,91]. Moreover after the combination of g-C₃N₄ and P3HT, the binding energy of N shifted positively, implying possible interactions between P3HT and g-C₃N₄, such as van der Waals interactions or electrostatic interactions [76,92]. Figure 5d shows the S 2p core-level XPS spectra of the P3HT and P3HT/g-C₃N₄ composite films. Two peaks were deconvoluted from the S 2p region, namely the S 2p^3/2^ and S 2p^1/2^ peaks centered at 165.58 and 166.78 eV, respectively. These peaks were associated with the C-S-C bond in P3HT. Owing to surface oxidation, a new binding energy peak was observed at 165.2 eV and was assigned to the sulfate in the P3HT/g-C₃N₄ composite films [89]. The binding energies of S 2p^3/2^ and S 2p^1/2^ shifted to 165.98 and 167.1 eV, respectively, suggesting a charge-transfer interaction between P3HT and g-C₃N₄ [92,93]. Figure 5e displays the O 1s core-level XPS spectra. In the case of g-C₃N₄, a weak O 1s peak was detected at 531.08 eV, which corresponded to the formation of N-C-O, most likely because the adsorbed water oxidized the sample surface; the peak at 532.48 eV was assigned to the adsorbed O atom [89,94]. The peaks of the P3HT/g-C₃N₄ composite films at 532.18 and 534.08 eV were assigned to the adsorbed O_2_ molecules and the formation of S-O owing to the partial surface oxidation of P3HT, respectively [93].

### 2.5. Charge Transport Properties of P3HT/g-C₃N₄ Composite Films

**Figure 6:**
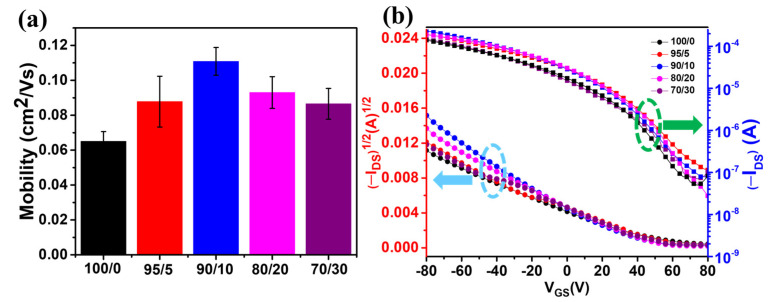
Comparison of (**a**) average charge carrier mobilities and (**b**) typical transfer curves of the bare P3HT (100/0), P3HT/g-C₃N₄ (95/5), P3HT/g-C₃N₄ (90/10), P3HT/g-C₃N₄ (80/20), and P3HT/g-C₃N₄ (70/30) OFETs.

The charge transport characteristics of the P3HT films hybridized with different amounts of g-C₃N₄ were studied by measuring the transfer curves of the P3HT/g-C₃N₄-based OFET devices with the bottom-gate bottom-contact geometry. The composite-film-based OFETs exhibited enhanced field effect mobilities (Figure 6a,b) compared to those of the bare P3HT film based OFETs. Specifically, as the g-C₃N₄ content was increased to up to 10 wt.%, the charge mobility of the composite films increased to up to ~1.1 × 10^−^^1^ cm^2^ V^−^^1^ S^−^^1^, which was approximately two times higher than that (6.5 × 10^−^^2^ cm^2^ V^−^^1^ S^−^^1^) of the bare P3HT films. This significant mobility enhancement was attributed to the enhanced formation of crystalline P3HT nanofibrillar aggregates upon g-C₃N₄ addition. However, the charge mobility of the hybrid devices containing more than 10 wt.% g-C₃N₄ decreased. Nonetheless, it was higher than the mobility of the bare P3HT film based OFETs. This decrease in charge mobility at higher g-C₃N₄ concentrations was anticipated owing to the significantly decreased film homogeneity and the increased density of grain boundaries between the crystal domains that do not contribute efficiently to the transportation pathways of the charge carriers [74,81,86]. Figure 6b presents the transfer characteristics of the devices, which indicate a typical P-channel OFET operation in the accumulation mode under a negative gate voltage [80].

### 2.6. Sensing Performance of P3HT/g-C₃N₄ Composite-Based OFET Sensors

**Figure 7:**
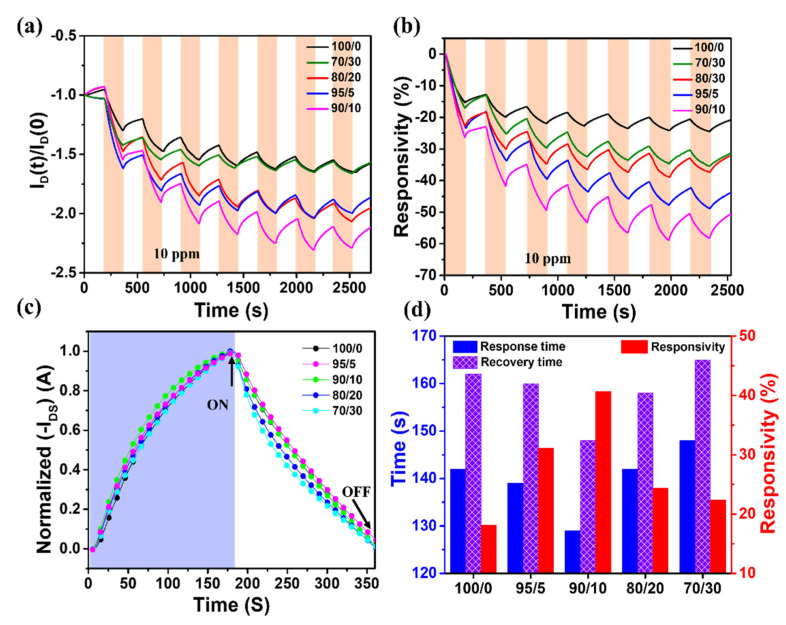
(**a**) Changes in the drain current of the P3HT/g-C₃N₄ film − based OFET sensors and (**b**) the real−time response of the corresponding OFET sensors upon exposure to 10 ppm NO and synthetic dry air. (**c**) Normalized drain currents and (**d**) corresponding response/recovery times (left axis) and responsivity (right axis) of the OFET sensors (V_DS_ = −40 V, V_GS_ = −10 V).

The NO gas sensing properties of the P3HT/g-C₃N₄ OFETs were repeatedly investigated by exposing the devices to 10 ppm NO gas for 180 s and then to synthetic dry air as the background atmosphere. The source-drain current (I_DS_) was measured at the drain voltage (V_D_) of −40 V and gate voltage (V_G_) of −10 V. Compared to the bare P3HT film based OFETs, which exhibited only a marginal change in the output current, the P3HT/g-C₃N₄ composite gas sensors showed significant variation in the drain current upon repeated exposure to 10 ppm NO gas (Figure 7a), resulting in improved responsivity to NO gas (Figure 7b). Because NO molecules act as oxidizing agents, they tend to withdraw electrons from the P3HT, which is a p-type semiconductor [3,95]. Therefore, the NO adsorbed by g-C₃N₄ can induce p-type doping. This increases the hole carrier density, resulting in an increase in the drain current of the P3HT/g-C₃N₄ composite OFETs [3,46,95,96,97]. Furthermore, when the P3HT/g-C₃N₄ sensors were exposed to 10 ppm NO gas for seven cycles, they exhibited good repeatability (Figure 7a,b), which is beneficial for their practical application. However, a slight decrease in responsivity was observed in the initial testing cycle owing to the incomplete desorption of the NO gas molecules from the P3HT film [23,73].

The sensing properties, namely responsivity, response time, and recovery time, of the P3HT/g-C₃N₄ OFET devices were investigated, as shown in Figure 7c,d. The responsivities of the P3HT/g-C₃N₄ composite film based OFET sensors were significantly higher than those of the bare P3HT film based OFET sensors (~18.1%). For instance, the responsivities of the OFET sensors containing 5, 10, 20, and 30 wt.% g-C₃N₄ were 31.1, 40.6, 24.3, and 22.4%, respectively. In particular, the responsivity of the P3HT/g-C₃N₄ (90/10) composite sensor was 2.2 times higher than that of the bare P3HT OFET sensor (Figure 7d). This increase in responsivity may be attributed to the following reasons: First, the addition of g-C₃N₄ provided a large surface area and abundant active sites for interaction with NO gas molecules, thereby enhancing the responsivity of the P3HT/g-C₃N₄ OFET sensors [98]. Second, the charge-transfer interaction between P3HT and g-C₃N₄ in the composite films led to the effective delivery of the NO-gas-generated charge carriers to the P3HT channels, which were created by the interaction of g-C₃N₄ with NO molecules. Third, because the P3HT/g-C₃N₄ composite sensors exhibited higher carrier mobilities, the gas-generated charge carriers were transported more effectively within the composite films compared to the bare P3HT films [46]. Consequently, the P3HT/g-C₃N₄ composite films possessed high NO gas conductivities, resulting in significant current variation and, thus, high sensing performance. Importantly, the responsivity of the OFET sensor based on P3HT/g-C₃N₄ with 10 wt.% of g-C₃N₄ was considerably higher than those of the devices based on the P3HT/g-C₃N₄ composite films with higher g-C₃N₄ concentrations (i.e., 20 and 30 wt.%). This degradation in sensing performance may be attributed to the existence of a large number of defects that impede efficient charge transport due to the inhomogeneity of the composite films [99]. As a result, the P3HT/g-C₃N₄ sensor presented a progressive tendency until g-C₃N₄ concentrations of 10 wt.%, which indicated the optimal mass percentage of g-C₃N₄ in the composite system.

The response and recovery times of the sensors were estimated by normalizing the drain current recorded during the exposure of the gas sensors to 10 ppm NO gas for 180 s, followed by purging with dry air for 180 s (Figure 7c,d). The P3HT/g-C₃N₄ (90/10) composite sensors presented excellent response and recovery times (129 and 148 s, respectively) compared to those of the bare P3HT sensors (142 and 162 s, respectively). The obtained response and recovery times of the P3HT/g-C₃N₄ composite sensors were at least ~26% and ~79%, respectively, shorter than those previously reported for organic NO sensors [4].

**Figure 8:**
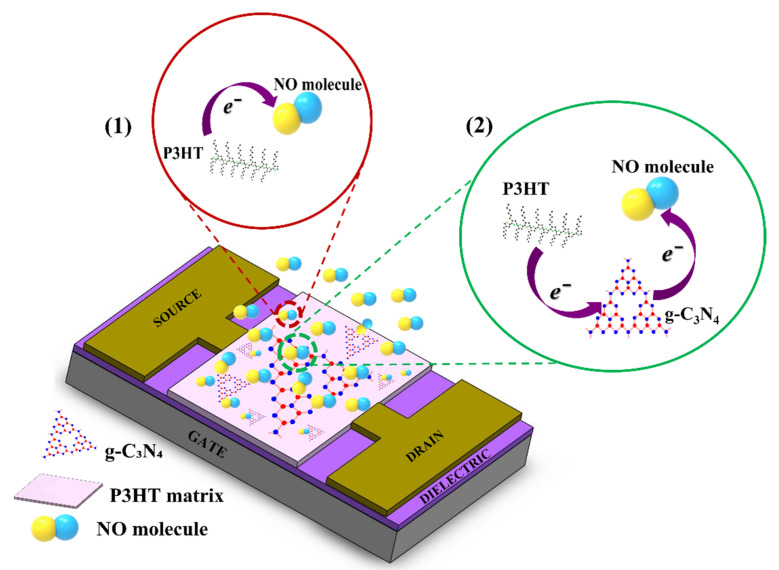
Schematic illustration of the gas sensing mechanism between (1) P3HT and NO molecules and (2) P3HT/g-C₃N₄ and NO molecules.

The gas sensing mechanism can be explained primarily on the basis of a charge transfer process, as shown in Figure 8. NO gas molecules can be recognized through two types of interactions, namely (1) P3HT-NO and (2) g-C₃N₄ -NO interactions. In the first type of interaction, the oxidizing NO gas withdraws electrons from P3HT owing to its strong electron affinity, thereby generating hole carriers in P3HT [95]. The generated hole carriers are transported through the P3HT matrix because of its superior hole transport capability.

In the second type of interaction, the adsorption of NO gas molecules on the g-C₃N₄ in the P3HT/g-C₃N₄ composite decreases the electron concentration in the g-C₃N₄ because the NO molecules withdraw electrons from the g-C₃N₄. Subsequently, electrons are transferred from P3HT to g-C₃N₄ for the restoration of the electron density of g-C₃N₄, resulting in the generation of hole carriers in P3HT. The hole carriers generated by both interactions are transported through the P3HT matrix, which in turn increases the current variation in the p-type P3HT/g-C₃N₄ OFET device. On the basis of the NO sensing results in Figure 7, the g-C₃N₄-NO interaction has a more dominant effect on the sensing performance of the P3HT/g-C₃N₄ OFET sensors compared to the P3HT-NO interaction. As previously mentioned, g-C₃N₄ has an excellent ability to detect gas molecules because it houses abundant active sites that can strongly interact with analytes. In summary, the synergistic effect of combining g-C₃N₄ and P3HT significantly improved the NO gas sensing performance of the fabricated conjugated-polymer-based OFET sensors because g-C_3_N_4_ provided abundant active sites for detecting NO gas and P3HT served as an efficient pathway for the gas-generated hole carriers.

**Figure 9:**
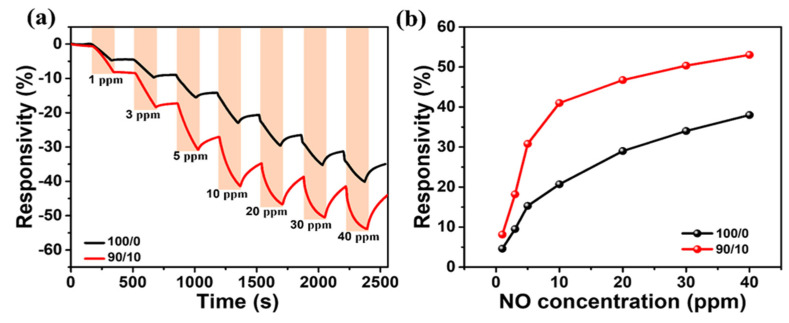
(**a**) Real−time responses of bare P3HT (100/0) and P3HT/g-C₃N₄ (90/10) OFET sensors as a function of time with dynamic NO concentration. (**b**) Responsivity plots of the corresponding sensors as a function of NO concentration.

We evaluated the dynamic responsivity of the bare P3HT (100/0) and P3HT/g-C₃N₄ (90/10) OFET sensors as a function of NO gas concentration in the range of 1 to 40 ppm with synthetic air gas as the background atmosphere. Each step consisted of 3 min of exposure to NO (adsorption) followed by 3 min of exposure to air (desorption). Figure 9a shows the real-time response curves of the bare P3HT (100/0) and P3HT/g-C₃N₄ (90/10) OFET sensors to different concentrations of NO gas. The response values of the two sensors increased as the NO gas concentration increased in the range of 1–40 ppm. Moreover, the response amplitude of the P3HT/g-C₃N₄ (90/10) OFET sensor was higher than that of the bare P3HT OFET sensor, and the difference between the response values of the two sensors increased as the NO concentration increased, indicating that the gas sensing performance of the proposed composite sensor was superior. Specifically, as the NO gas concentration increased from 0 to 10 ppm, the responsivities of the bare P3HT sensor (100/0) and P3HT/g-C₃N₄ (90/10) sensor increased significantly from 4.6 to 18.1% and from 8.1 to 40.6%, respectively. When the NO concentration was further increased to up to 40 ppm, the responsivity of the bare P3HT OFET sensor increased gradually (38%), whereas that of the P3HT/g-C₃N₄ OFET sensor increased relatively mildly (53%) at a slow rate. This decreased rate of the increase in responsivity may be ascribed to the nearly occupied adsorption sites in the composite OFET sensors [46]. The sensing parameters obtained in this study are presented in Table 1 and compared to those of metal-oxide-based sensors presented in previous studies [100,101,102]. This result indicates that the sensing parameters of the P3HT/g-C₃N₄ OFET sensor are superior to those of the conjugated polymer film based sensors and comparable to those of the metal-oxide-based sensors.

**Figure 10:**
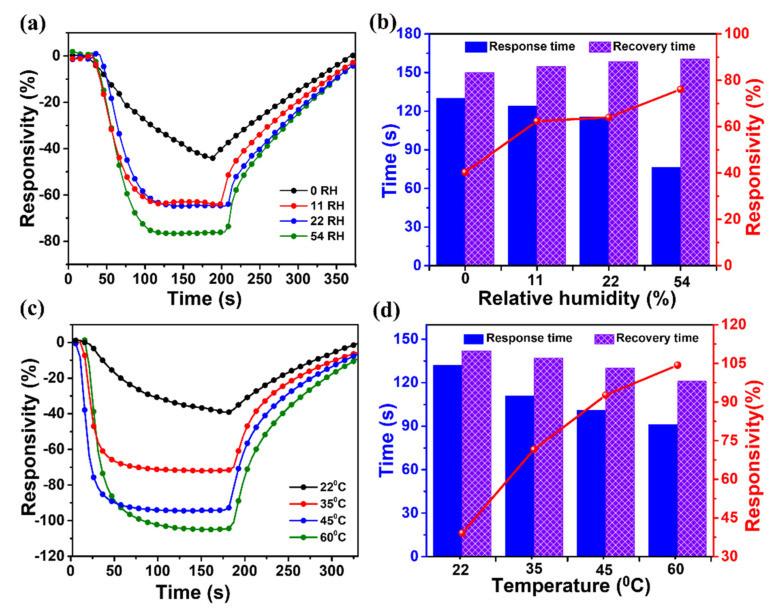
(**a**) Time-dependent responsivity and (**b**) corresponding response/recovery time and responsivity values of the OFET sensors based on P3HT/g-C₃N₄ (90/10) films upon exposure to 10 ppm NO and bare synthetic air under different RHs. (**c**) Time −dependent responsivity and (**d**) corresponding response/recovery time and responsivity values of the OFET sensors based on the P3HT/g-C₃N₄ (90/10) films at different temperatures (°C).

In practical settings, humidity and temperature substantially affect the gas-detection behaviors of sensors. Additionally, water molecules in humid environments affect the selective identification of NO by sensors. Water molecules can diffuse into the OSC layer or the interface between the OSC layer and the dielectric layer to form donor- and acceptor-like traps that degrade device performance [104,105]. Therefore, we investigated the effect of humidity on the response of the P3HT/g-C₃N₄ OFET sensor by varying the RH, as shown in Figure 10a,b. In this evaluation, the sensor was exposed to 10 ppm NO gas under different RH conditions (i.e., 0%, 11%, 22%, and 54%). To achieve a steady-state baseline, the chamber in which the sensor was placed was purged with humidified air at a specific RH before introducing the NO gas. This was required to prevent sensor response fluctuations due to changes in the RH of the input gas. The desired RH was generated by mixing the NO gas with humidified air, and the mixture was then injected into the sensor chamber. As the RH increased from 0 to 54%, the responsivity of the composite sensor gradually increased from 40.3% to 76.1% (Figure 10a,b). This increase was attributed to the competitive adsorption of water molecules and NO molecules onto the surface of the P3HT/g-C₃N₄ composite films, resulting in a limited adsorption of NO molecules at high RHs [106,107,108]. Note that P3HT was oxidized by the water molecules adsorbed on the surface of the composite films, which increased the range of the drain current variation of the composite sensor [109,110]. It was found that the response time of the P3HT/g-C₃N₄ OFET sensor was generally faster under high RHs; the response time decreased from 129 s to 76 s as the RH increased from 0 to 54% (Figure 10b). This decrease was attributed to the fact that water molecules were adsorbed faster than NO gas molecules on the surface of the composite sensor [111]. Intriguingly, the recovery time remained almost constant as the RH increased, indicating similar desorption rates for water and NO molecules.

Temperature is another crucial environmental factor that must be considered in sensor operation. The sensing behavior of the P3HT/g-C₃N₄ composite sensor was significantly influenced by the operating temperature, as shown in Figure 10c,d. Notably, the responsivity of the composite sensor increased as the temperature increased. For instance, the responsivity values calculated at 22, 35, 45, and 60 °C were 40.6, 71.6, 92.7, and 104.3%, respectively. This enhanced performance was attributed to the thermal excitation of g-C_3_N_4_, which introduced an abundance of high-energy carriers that enhanced the surface interactions between NO gas molecules and the P3HT/g-C_3_N_4_ composite films. Moreover, the response time decreased significantly by 31% as the gas temperature increased from 22 °C to 60 °C. The significant reduction was attributed to the rapid diffusion of gas molecules at higher temperatures [62,99,112].

## 3. Materials and Methods

### 3.1. Materials and Chemicals

Regioregular P3HT (regioregularity ≈ 96% and Mw ≈ 51 kDa) was purchased from Rieke Metals Inc. (Lincoln, NE, USA). Chloroform (anhydrous-grade) was purchased from Sigma-Aldrich Co (St. Louis, MO, USA). All chemicals were used without further purification. g-C_3_N_4_ was synthesized and purified by following a previously reported method [113].

### 3.2. Preparation of P3HT/g-C_3_N_4_ Composite Solutions

P3HT, used as the model conjugated polymer, was dissolved in chloroform at a concentration of 10 mg/2 mL and stirred for 60 min at 55 °C to obtain a homogeneous P3HT solution [74,114]. Alternatively, a relevant amount of g-C_3_N_4_ was added to 2 mL of chloroform, and the solution was ultrasonicated for 20 min [115] to ensure the good dispersion of g-C_3_N_4_ in it. After cooling the two solutions to room temperature, they were mixed at appropriate weight ratios (P3HT: g-C_3_N_4_ = 95:5, 90:10, 80:20, and 70:30) at a concentration of 10 mg/2 mL in 20 mL borosilicate glass vials to obtain P3HT/g-C_3_N_4_ composite solutions. These composite solutions were heated at 55 °C to ensure homogenous dissolution and dispersion, followed by cooling to room temperature. Furthermore, the composite solutions were photoirradiated by exposing them to UV light (310 μW cm^−2^, 254 nm) for 7 min under gentle stirring, using a magnetic stirrer to improve the dispersion stability of the g-C_3_N_4_ [73,74].

### 3.3. Fabrication of OFET Devices Based on P3HT/g-C_3_N_4_ Composite Solutions

OFET devices with bottom-gate bottom-contact geometry (channel length = 50 μm, width = 2000 μm) were fabricated by following the procedure described in [74,116,117,118]. The source and drain electrodes were deposited on SiO_2_/Si substrates using a thermal evaporator (JVMS-23M151S) at the Energy Convergence Core Facility at Chonnam National University. The FET substrates were cleaned by means of sonication in acetone for 15 min, after which they were rinsed with acetone, methanol, and isopropanol. The FET substrates were then treated in a UV-ozone cleaner for 30 min to remove any residual organic contaminants before the film deposition process. The P3HT/g-C_3_N_4_ composite films were fabricated by spin-coating the corresponding solutions onto the precleaned FET device substrates at a spin rate of 2000 rpm for 60 s under ambient conditions and annealed at 55 °C in a vacuum oven overnight to remove residual solvents from the films.

### 3.4. OFET Characterization and Gas Sensor Test

The UV-Vis absorption spectra of the P3HT/g-C_3_N_4_ composite films were recorded using a UV-Vis spectrometer (Evolution 220, Themo Scientific, Daejeon, South Korea). Thin composite films were spin-coated on precleaned glass substrates for the OFET measurements by following the procedures used for the OFET device fabrication. The dispersion of g-C_3_N_4_ in the P3HT/g-C_3_N_4_ composite solution was visualized by means of scanning electron microscopy (SEM; JSM-7900F, Jeol, Tokyo, Japan) and optical microscopy (Leica DM750, Leica Microsystems, Wetzlar, Germany). The surface morphologies of the P3HT/g-C_3_N_4_ composite films were imaged using an atomic force microscope (AFM; NX20, Park systems, Suwon, South Korea) equipped with a silicon tip operated in the tapping mode. Raman spectroscopy (Renishaw Invia, 514 laser, Nanobase Seoul, South Korea) was used to evaluate the structural features of the prepared composite films. The XPS spectra of the films were recorded using a spectrometer (K-Alpha +, Thermo Fisher Scientific) equipped with an Al kα X-ray line to analyze the surfaces of the films. The electrical properties of the composite films were characterized using a semiconductor parameter analyzer (Keithley 4200, Keithley Instruments, LLC, Cleveland, Ohio, USA) in an N_2_-filled glovebox. The charge mobility of the films was calculated from the saturation regime (drain-source voltage (V_DS_) = −80 V) by following an established research approach [23,81,118].

The NO gas sensing test of the fabricated P3HT/g-C_3_N_4_ OFET sensor was conducted using a homemade gas-detection system equipped with volumetric flow controllers and a data acquisition system. NO gas and synthetic dry air were injected into a Teflon chamber through volumetric flowmeters at a fixed flow rate of 100 mL/min. A standard NO gas mixture (1000 ppm in synthetic dry air) was diluted using synthetic dry air to generate gas mixtures with various NO concentrations. For the sensor humidity tests, the gas stream was humidified by bubbling synthetic dry air in water and combining it with NO gas at a suitable ratio. The temperature of the gas stream was controlled by dipping the gas tube connected to the chamber housing the sensor devices in a water bath maintained at various temperatures. The real-time variation in the source-drain current (I_DS_) of the OFET sensors was recorded using a semiconductor parameter analyzer under V_GS_ = −10 V and V_DS_ = −40 V at 3 min intervals. The sensing responsivity (R) of the fabricated sensor was computed using the following equation [119]:

(2) $$R\left(\%\right)=\frac{I_{\mathrm{NO}}-I_{\mathrm{air}}}{I_{\mathrm{air}}}\times 100\%$$

where I_NO_ and I_air_ are the real-time drain current values of the OFET sensor upon exposure to NO gas and synthetic dry air, respectively. The response time (defined as the time required by the sensor to reach 90% of its steady-state response value after its exposure to a given gas concentration) and recovery time (similarly defined as the time required by the sensor to decrease to 10% of its steady-state response value) of the fabricated OFET sensors were computed [111].

## 4. Conclusions

In this study, we reported an OFET sensor based on P3HT films incorporated with graphitic carbon nitride for detecting NO gas. The effective gas-capturing property of g-C₃N₄ allowed it to function as a gas-adsorbing site in the polymer matrix, resulting in improved sensing performance. As a result, the sensing performance of the P3HT/g-C₃N₄ composite film based OFET sensors was superior to that of the bare P3HT sensor. Specifically, the sensor based on the P3HT/g-C₃N₄ (90/10) composite films exhibited outstanding sensing ability when exposed to 10 ppm NO gas in terms of responsivity (40.6%), response time (129 s), and recovery time (148 s) vs. 18.1%, 142 s, and 162 s, respectively, compared to the bare P3HT sensors. The sensing performance was discovered to be governed by the charge-transfer interactions between P3HT and g-C₃N₄; the large surface area of g-C₃N₄, which provides abundant active sites for effective gas analyte interactions; and higher carrier mobilities that facilitate the effective transport of the gas-generated charge carriers within the composite films, as opposed to the bare P3HT films. These results highlighted the ability of g-C₃N₄ to enhance the sensing performance of conjugated-polymer-based OFET gas sensors. We believe that the synthesized P3HT/g-C_3_N_4_ composite films presented herein will offer new opportunities for sustainable applications in gas sensing, especially for the detection of other potentially harmful gases such as SO_2_, NO_2_, and NH_3_ and volatile organic compounds such as methanol, ethanol, and acetone.

## Figures and Tables

**Table 1:** Comparison of the NO sensing performances of the prepared P3HT/g-C_3_N_4_ sensors and other sensors reported in the literature.

Materials	Device Type	NO Concentration (ppm)	NO Response (%)	Response Time (s)	Recovery Time (s)	Ref.
P3HT/g-C_3_N_4_	OFET	10	40.6	129	148	This work
PEDOT:PSS	Resistor	10	2.2	527	1780	[103]
PBDTTT-C-T	VOD	0.01	1.3	60	-	[3]
DPP-DTT	OFET	0.01–10	150.3	174–267	693	[4]
PCDTBT	Resistor	100	80.6	300	2100	[95]
Coralline-like porous ZnO	Resistor	40	23.59	331	1285	[102]
Cu^2+^/PANI/WO_3_	SAW	0.01	-	97	36	[10]
Pt/In_2_O_3_–WO_3_	Resistor	1000	23.9	750	918	[101]
Ag@plate-WO_3_	Resistor	5	1.59	600	600	[100]

VOD: vertical organic diode, SAW: surface acoustic wave.

## Data Availability

The datasets generated and/or analyzed during this study are available upon reasonable request from the corresponding author.

## Supplementary Materials

The following supporting information can be downloaded at https://www.mdpi.com/article/10.3390/ijms24021158/s1.
