# Supplementary material for: Enhanced Nitric Oxide Sensing Performance of Conjugated Polymer Films through Incorporation of Graphitic Carbon Nitride
Source: Int J Mol Sci. 2023 Jan 6;24(2):1158. doi: 10.3390/ijms24021158 (PMC9864893; doi:10.3390/ijms24021158)
Supplement: Supplementary file 1 [file ijms-24-01158-s001.zip › Kyokunzire et al_Supporting Information.pdf]

Supporting Information

Enhanced Nitric Oxide Sensing Performance Based on Conjugated Polymer Films Incorporated with Graphitic Carbon Nitride

Proscovia Kyokunzire[1], *Ganghoon Jeong*[1], *Seo Young Shin*[1], *Hyeong Jun Cheon*[1], *Eunsol Wi*[1], *Minhong Woo*[1], *Trang Thi Vu*[2], *and Mincheol Chang*[1,2,3,]*

[1]Department of Polymer Engineering, Graduate School, Chonnam National University, Gwangju 61186, South Korea

[2]Alan G. MacDiarmid Energy Research Institute, Chonnam National University, Gwangju 61186, South Korea

[3]School of Polymer Science and Engineering, Chonnam National University, Gwangju 61186, South Korea

*Correspondence: mchang35@chonnam.ac.kr

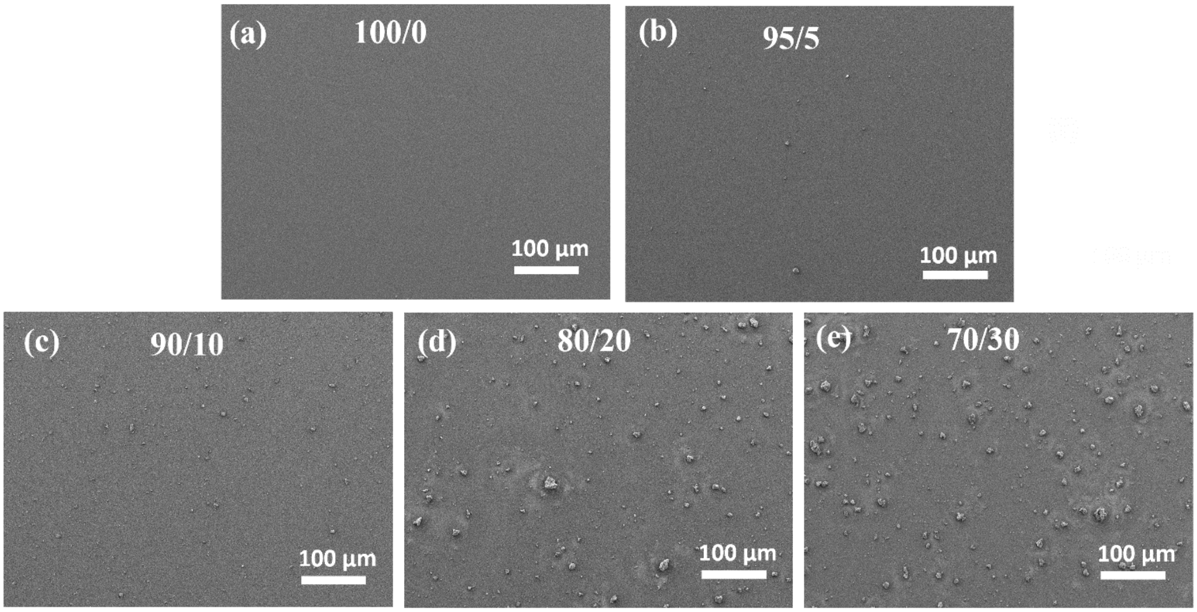

Figure S1. Scanning Electron Microscopy (SEM) images of films deposited from composite solutions; (a) bare P3HT (100/0) (b) P3HT/ g-C_3N_4 (95/5), (c) P3HT/ g-C_3N_4 (90/10), (d) P3HT/ g-C_3N_4 (80/20 and (e) P3HT/ g-C_3N_4 (70/30)

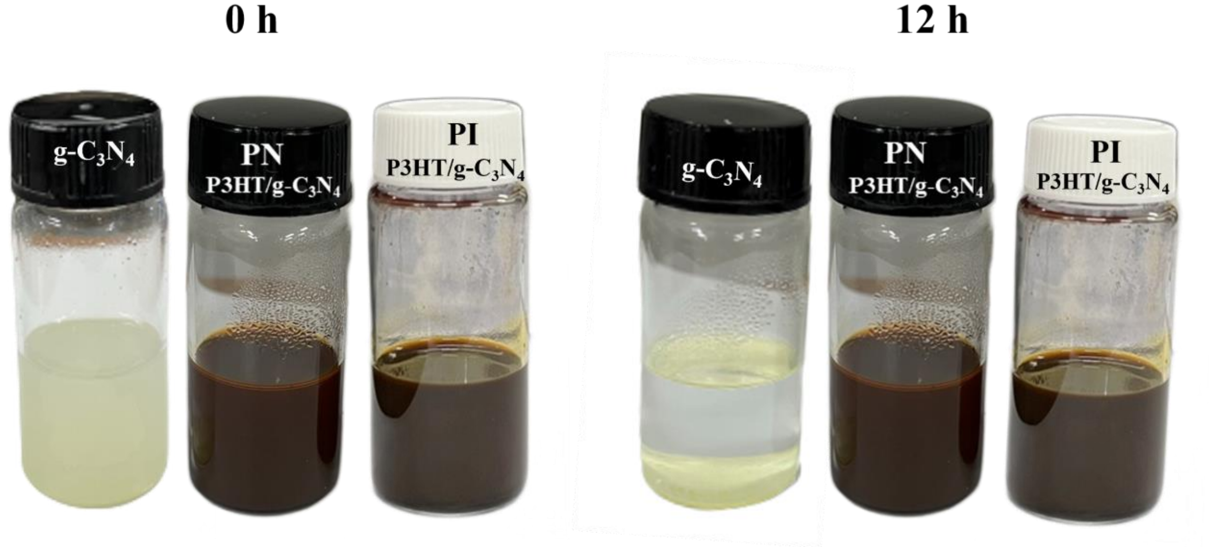

Figure. S2. Photographs of g-C_3N_4, pristine P3HT/g-C_3N_4, and photoirradiated P3HT/g-C_3N_4 composite solution placed for 0 and 12 h after preparation.

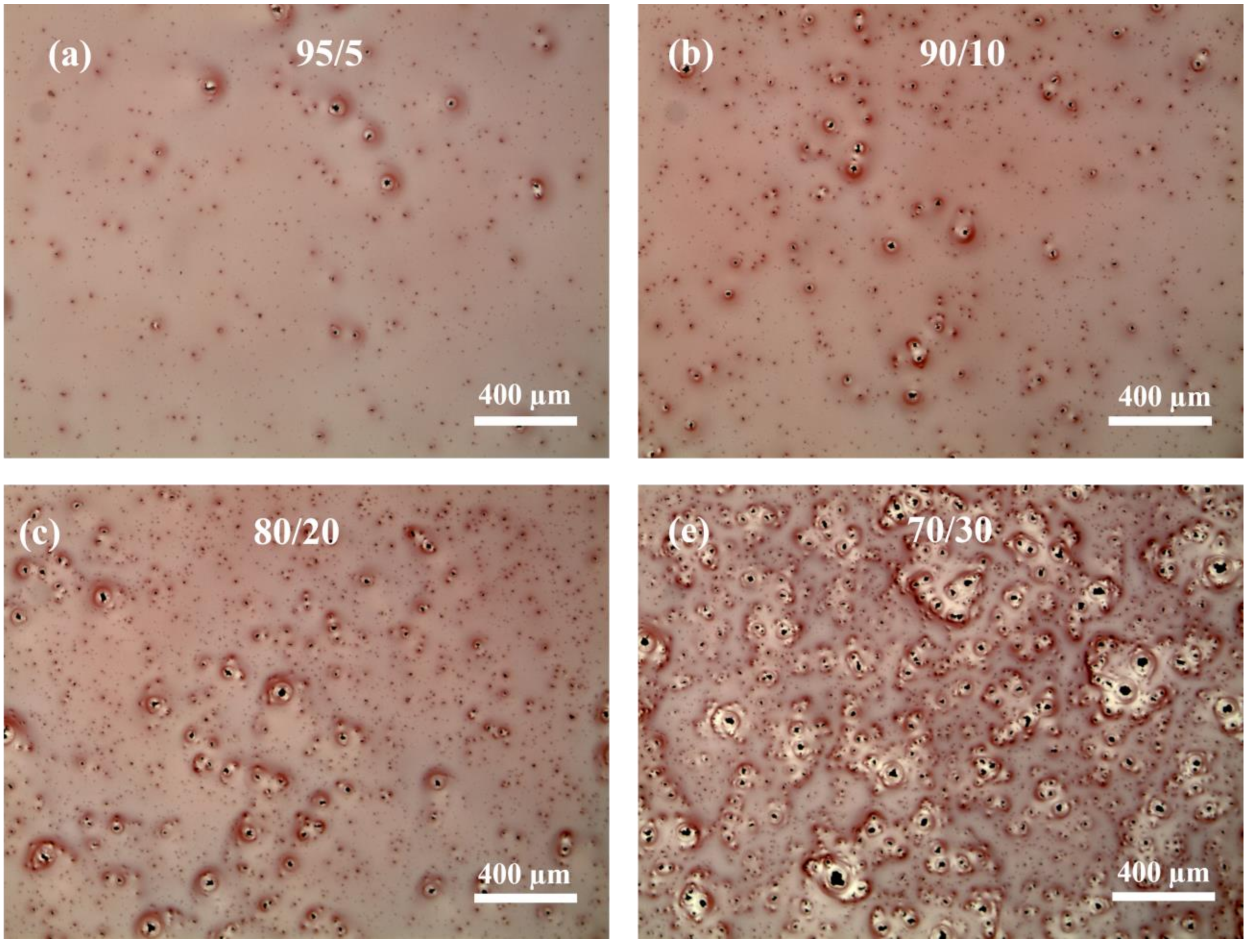

Figure. S3. OM images of films deposited using the pristine (a) P3HT/g-C_3N_4 (95/5), (b) P3HT/g-C_3N_4 (90/10), (c) P3HT/g-C_3N_4 (80/20), and (d) P3HT/g-C_3N_4 (70/30) composite solutions.
